# *Steccherinum tenuissimum* and *S*. *xanthum* spp. nov. (Polyporales, Basidiomycota): New species from China

**DOI:** 10.1371/journal.pone.0244520

**Published:** 2021-01-13

**Authors:** Ya-Xing Wu, Jian-Rong Wu, Chang-Lin Zhao

**Affiliations:** 1 Key Laboratory for Forest Resources Conservation and Utilization in the Southwest Mountains of China, Ministry of Education, Southwest Forestry University, Kunming, P. R. China; 2 College of Biodiversity Conservation, Southwest Forestry University, Kunming, P. R. China; Beijing Forestry University, CHINA

## Abstract

Two new wood-inhabiting fungal species, *Steccherinum tenuissimum* and *S*. *xanthum* spp. nov. are described based on a combination of morphological features and molecular evidence. *Steccherinum tenuissimum* is characterized by an annual growth habit, resupinate basidiomata with an odontioid hymenial surface, a dimitic hyphal system with clamped generative hyphae, strongly encrusted cystidia and basidiospores measuring 3–5 × 2–3.5 μm. *Steccherinum xanthum* is characterized by odontioid basidiomata and a monomitic hyphal system with generative hyphae bearing clamp connections and covering by crystals, colourless, thin-walled, smooth, IKI–, CB–and has basidiospores measuring 2.7–5.5 × 1.8–4.0 μm. Sequences of the ITS and nLSU nrRNA gene regions of the studied samples were generated, and phylogenetic analyses were performed with maximum likelihood, maximum parsimony and Bayesian inference methods. The phylogenetic analyses based on molecular data of ITS + nLSU sequences showed that two new *Steccherinum* species felled into the residual polyporoid clade. Further investigation was obtained for more representative taxa in *Steccherinum* based on ITS + nLSU sequences, which demonstrated that *S*. *tenuissimum* and *S*. *xanthum* were sister to *S*. *robustius* with high support (100% BP, 100% BS and 1.00 BPP).

## Introduction

*Steccherinum* Gray (Steccherinaceae, Polyporales) is typified by *S*. *ochraceum* (Pers. ex J.F. Gmel.) Gray, and this genus is characterized by resupinate to effused-reflexed or pileate basidiome with a membranaceous consistency, odontioid to hydnoid hymenophore, a monomitic or dimitic hyphal system with clamped or simple-septate generative hyphae, subclavate to clavate basidia and basidiospores that are colourless, thin-walled, smooth, ellipsoid to subcylindrical, acyanophilous and negative to Melzer’s reagent [1, 2]. To date, approximately 40 species have been accepted in the genus worldwide [3].

Molecular studies related to *Steccherinum* have been carried out [4–8]. Larsson [4] analysed the classification of corticioid fungi, and suggested that *S*. *ochraceum* was nested in the Meruliaceae and grouped with *Junghuhnia nitida* (Pers.) Ryvarden. The phylogeny of the poroid and hydnoid genera *Antrodiella* Ryvarden & I. Johans., *Junghuhnia* Corda, and *Steccherinum* (Polyporales, Basidiomycota) was studied utilizing sequences of the gene regions ITS, nLSU, mtSSU, atp6, rpb2, and tef1, which revealed that the genus *Steccherinum* was shown to contain both hydnoid and poroid species, and the taxa from *Junghuhnia* and *Steccherinum* grouped together mixed within *Steccherinum* clade [5]. A molecular study based on multi-gene datasets demonstrated that *Steccherinum* belonged to the residual polyporoid clade and the generic type (*S*. *ochraceum*) was grouped with *J*. *nitida* [6]. A revision of the family-level classification of the Polyporales, including eighteen families, showed that, *Steccherinum* was grouped with *Cerrena* Grey and *Panus* Fr. [7]. On the basis of a re-evaluation of *Junghuhnia* s. lat. based on morphological and multi-gene analyses, a new species, *Steccherinum neonitidum* Westphalen & Tomšovský, and three new combinations, *S*. *meridionale* (Rajchenb.) Westphalen, Tomšovský & Rajchenberg, *S*. *polycystidiferum* (Rick) Westphalen, Tomšovský & Rajchenb. and *S*. *undigerum* (Berk. & M.A. Curtis) Westphalen & Tomšovský, were introduced and *S*. *robustius* (J. Erikss. & S. Lundell) J. Erikss. grouped with *J*. *crustacea* (Jungh.) Ryvarden [8].

During investigations on wood-inhabiting fungi in southern China, two taxa which could not be assigned to any described species of *Steccherinum*, were found. To confirm the placement of the undescribed species in this genus, morphological examination and phylogenetic analyses based on the internal transcribed spacer (ITS) regions and the large subunit nuclear ribosomal RNA gene (nLSU) sequences were carried out.

## Materials and methods

### Morphological studies

The specimens studied are deposited at the herbarium of Southwest Forestry University (SWFC), Kunming, Yunnan Province, P.R. China. The macromorphological descriptions are based on field notes. The colour terms follow Petersen [9]. Micromorphological data were obtained from the dried specimens, and observed under a light microscope (Nikon Eclipse E 100, Tokyo, Japan) following a previous study [10]. The following abbreviations were used for the microscopic characteristic descriptions: KOH = 5% potassium hydroxide, CB = cotton blue, CB– = acyanophilous, IKI = Melzer’s reagent, IKI– = both non-amyloid and non-dextrinoid, L = mean spore length (arithmetic average of all spores), W = mean spore width (arithmetic average of all spores), Q = variation in the L/W ratios between the specimens studied, n (a/b) = number of spores (a) measured from given number (b) of specimens.

### Molecular procedures and phylogenetic analyses

The CTAB rapid plant genome extraction kit DN14 (Aidlab Biotechnologies Co., Ltd, Beijing) was used to obtain genomic DNA from dried specimens, according to the manufacturer’s instructions, with some modifications: a small piece of dried fungal specimen (approximately 30 mg) was ground to a powder with liquid nitrogen. The powder was transferred to a 1.5 mL centrifuge tube, suspended in 0.4 mL of lysis buffer, and incubated in a 65°C water bath for 60 min. Then, 0.4 mL of phenol-chloroform (24:1) was added to the tube, and the suspension was shaken vigorously. After centrifugation at 13,000 rpm for 5 min, 0.3 mL of supernatant was transferred to a new tube and mixed with 0.45 mL of binding buffer. The mixture was then transferred to an adsorbing column (AC) for centrifugation at 13,000 rpm for 0.5 min. Then, 0.5 mL of inhibitor removal fluid was added to the AC, and the solution was centrifuged at 12,000 rpm for 0.5 min. After washing twice with 0.5 mL of washing buffer, the AC was transferred to a clean centrifuge tube, and 0.1 mL of elution buffer was added to the middle of the adsorbed film to elute the genomic DNA. The ITS region was amplified with primer pairs ITS5 and ITS4 [11]. The PCR procedure for the ITS region was as follows: initial denaturation at 95°C for 3 min; followed by 35 cycles at 94°C for 40 s, 58°C for 45 s and 72°C for 1 min; and a final extension at 72°C for 10 min. The PCR products were purified and directly sequenced at Kunming Tsingke Biological Technology Limited Company. All newly generated sequences were deposited in GenBank (Table 1).

**Table 1:** List of species, specimens, and GenBank accession number of sequences used in this study.

Species name	Sample no.	GenBank accession no.		References
		ITS	nLSU	
*Abortiporus biennis*	EL 6503	JN649325	JN649325	[15]
*Antrodia albida*	CBS 308.82	DQ491414	DQ491414	[16]
*A*. *heteromorpha*	CBS 200.91	DQ491415	DQ491415	[16]
*Antrodiella semisupina*	X 242	JN710521	JN710521	[5]
*Byssomerulius corium*	FP 102382	KP135007	KP135230	[17]
*Ceriporiopsis gilvescens*	BRNM 710166	FJ496684	FJ496684	[18]
*Climacocystis borealis*	KH 13318	JQ031126	JQ031126	[6]
*Coriolopsis caperata*	LE(BIN) 0677	AB158316	AB158316	[18]
*Daedalea quercina*	Miettinen 12662	JX109855	JX109855	[6]
*Earliella scabrosa*	PR 1209	JN165009	JN165009	[19]
*Fomitopsis pinicola*	CCBAS 536	FJ608588	—	[20]
*F*. *rosea*	ATCC 76767	DQ491410	DQ491410	[16]
*Fragiliporia fragilis*	Dai 13080	KJ734260	KJ734260	[21]
*F*. *fragilis*	Dai 13559	KJ734261	KJ734261	[21]
*F*. *fragilis*	Dai 13561	KJ734262	KJ734262	[21]
*Ganoderma lingzhi*	Wu 100638	JQ781858	—	[22]
*Gelatoporia subvermispora*	BRNU 592909	FJ496694	FJ496694	[18]
*Grammothelopsis subtropica*	Cui 9035	JQ845094	JQ845097	[22]
*Heterobasidion annosum*	PFC 5252	KC492906	KC492906	[6]
*Hornodermoporus martius*	MUCL 41677	FJ411092	FJ411092	[23]
*Hypochnicium lyndoniae*	NL 041031	JX124704	JX124704	[6]
*Irpex lacteus*	DO 421/951208	JX109852	JX109852	[6]
*Junghuhnia crustacea*	X 262	JN710553	JN710553	[5]
*Mycoacia fuscoatra*	KHL 13275	JN649352	JN649352	[15]
*M*. *nothofagi*	KHL 13750	GU480000	GU480000	[24]
*Obba rivulosa*	KCTC 6892	FJ496693	FJ496693	[18]
*O*. *valdiviana*	FF 503	HQ659235	HQ659235	[25]
*Perenniporia medulla-panis*	MUCL 43250	FJ411087	FJ411087	[23]
*P*. *ochroleuca*	MUCL 39726	FJ411098	FJ411098	[23]
*P*. *chrysocreas*	KUC 2012112324	KJ668482	KJ668482	[17]
*Phlebia fuscotuberculata*	CLZhao 10239	MT020760	MT020738	[26]
*P*. *hydnoidea*	HHB 1993	KY948778	KY948778	[7]
*P*. *radiata*	AFTOL 484	AY854087	AY854087	[27]
*P*. *tomentopileata*	CLZhao 9509	MT020762	MT020740	[26]
*P*. *tongxiniana*	CLZhao 5217	MT020778	MT020756	[26]
*P*. *tremellosa*	ES 20082	JX109859	JX109859	[6]
*Piloporia sajanensis*	Mannine 2733a	HQ659239	HQ659239	[18]
*Podoscypha venustula*	LR 40821	JX109851	JX109851	[6]
*Polyporus tuberaster*	CulTENN 10197	AF516596	AF516596	[6]
*Sebipora aquosa*	Miettinen 8680	HQ659240	HQ659240	[25]
*Skeletocutis amorpha*	Miettinen 11038	FN907913	FN907913	[18]
*S*. *jelicii*	H 6002113	FJ496690	FJ496690	[18]
*S*. *portcrosensis*	LY 3493	FJ496689	FJ496689	[18]
*S*. *subsphaerospora*	Rivoire 1048	FJ496688	FJ496688	[18]
*Steccherinum autumnale*	VS 2957	JN710549	JN710549	[5]
*S*. *bourdotii*	RS 10195	JN710584	JN710584	[5]
*S*. *collabens*	KHL 11848	JN710552	JN710552	[5]
*S*. *fimbriatellum*	OM 2091	JN710555	JN710555	[5]
*S*. *fimbriatum*	KHL 11905	JN710530	JN710530	[5]
*S*. *formosanum*	TFRI 652	EU232184	EU232268	[8]
*S*. *lacerum*	TN 8246	JN710557	JN710557	[5]
*S*. *meridionalis*	MR 10466	KY174994	KY174994	[8]
*S*. *meridionalis*	MR 11086	KY174993	KY174993	[8]
*S*. *meridionalis*	MR 284	KY174992	KY174992	[8]
*S*. *neonitidum*	MCW 371/12	KY174990	KY174990	[8]
*S*. *neonitidum*	RP 79	KY174991	KY174991	[8]
*S*. *nitidum*	KHL 11903	JN710560	JN710560	[5]
*S*. *nitidum*	MT 33/12	KY174989	KY174989	[8]
*S*. *nitidum*	FP 105195	KP135323	KP135227	[17]
*S*. *ochraceum*	KHL 11902	JN710590	JN710590	[5]
*S*. *polycystidiferum*	RP 140	KY174996	KY174996	[8]
*S*. *polycystidiferum*	MCW 419/12	KY174995	KY174995	[8]
*S*. *pseudozilingianum*	MK 1004	JN710561	JN710561	[5]
*S*. *robustius*	G 1195	JN710591	JN710591	[5]
*S*. *tenue*	KHL 12316	JN710598	JN710598	[5]
*S*. *tenuispinum*	OM 8065	JN710599	JN710599	[5]
*S*. *tenuispinum*	LE 231603	KM411452	KM411452	[8]
*S*. *tenuispinum*	VS 2116	JN710600	JN710600	[5]
*S*. *tenuissimum*	CLZhao 894	MW204581	MW204570	this study
*S*. *tenuissimum*	CLZhao 3153	MW204582	MW204571	this study
*S*. *tenuissimum*	CLZhao 4294	MW204583	MW204572	this study
*S*. *tenuissimum*	CLZhao 5100	MW204584	MW204573	this study
*S*. *undigerum*	MCW 426/13	KY174986	KY174986	[8]
*S*. *undigerum*	MCW 472/13	KY174987	KY174987	[8]
*S*. *undigerum*	MCW 496/14	KY174988	KY174988	[8]
*S*. *xanthum*	CLZhao 4381	MW204585	MW204574	this study
*S*. *xanthum*	CLZhao 4479	MW204586	MW204575	this study
*S*. *xanthum*	CLZhao 5024	MW204587	MW204576	this study
*S*. *xanthum*	CLZhao 5030	MW204588	MW204577	this study
*S*. *xanthum*	CLZhao 5032	MW204589	MW204578	this study
*S*. *xanthum*	CLZhao 5044	MW204590	MW204579	this study
*S*. *xanthum*	CLZhao 8124	MW204591	MW204580	this study
*Stereum hirsutum*	NBRC 6520	AB733150	AB733325	[18]
*Tyromyces chioneus*	Cui 10225	KF698745	KF698745	[28]

**Fig 1:**
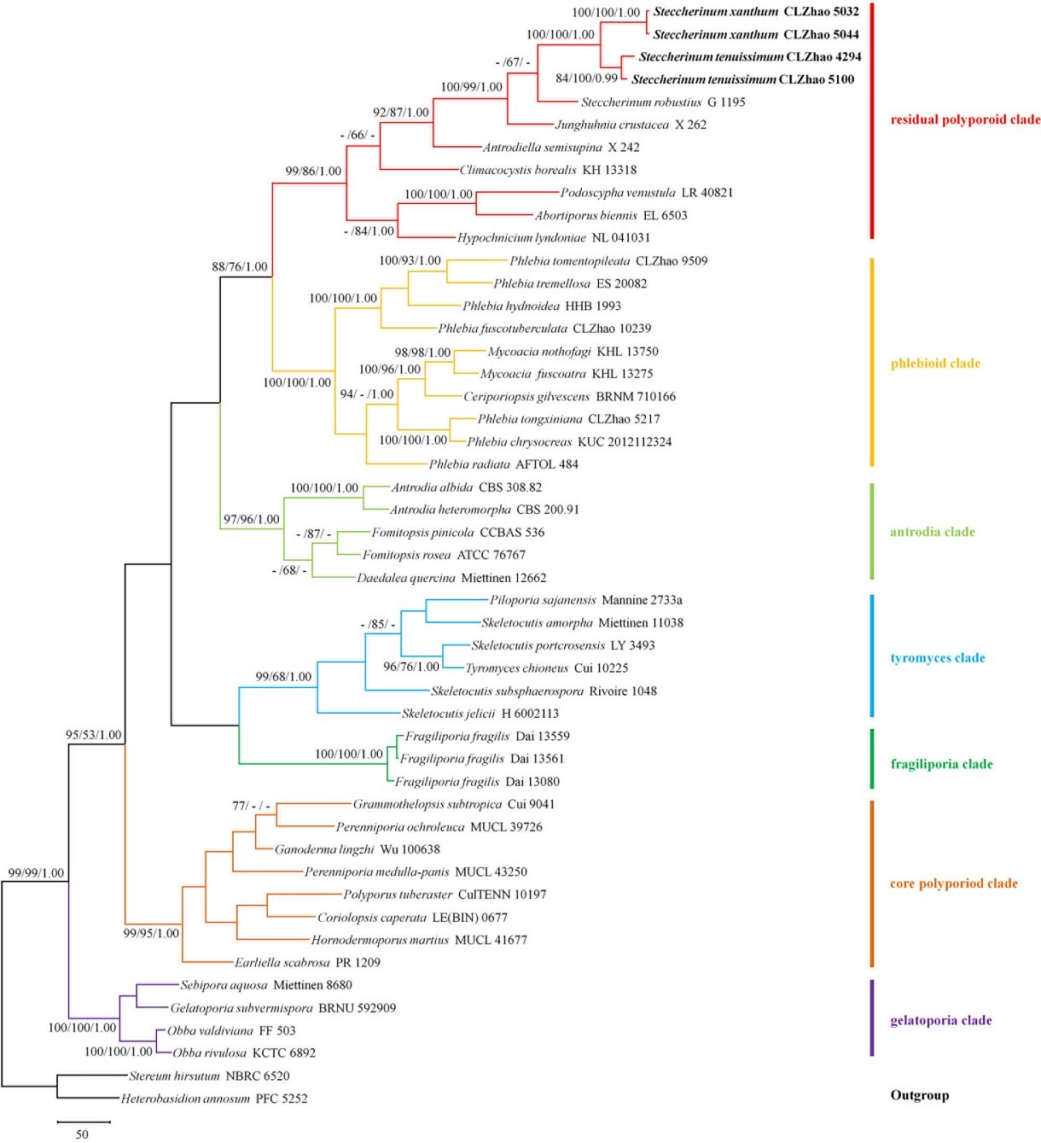
Maximum parsimony strict consensus tree illustrating the phylogeny of two new species and related species in Polyporales based on ITS + nLSU sequences. Branches are labelled with maximum likelihood bootstrap higher than 70%, parsimony bootstrap proportions higher than 50% and Bayesian posterior probabilities more than 0.95 respectively. Clade names follow Binder et al. [6].

**Fig 2:**
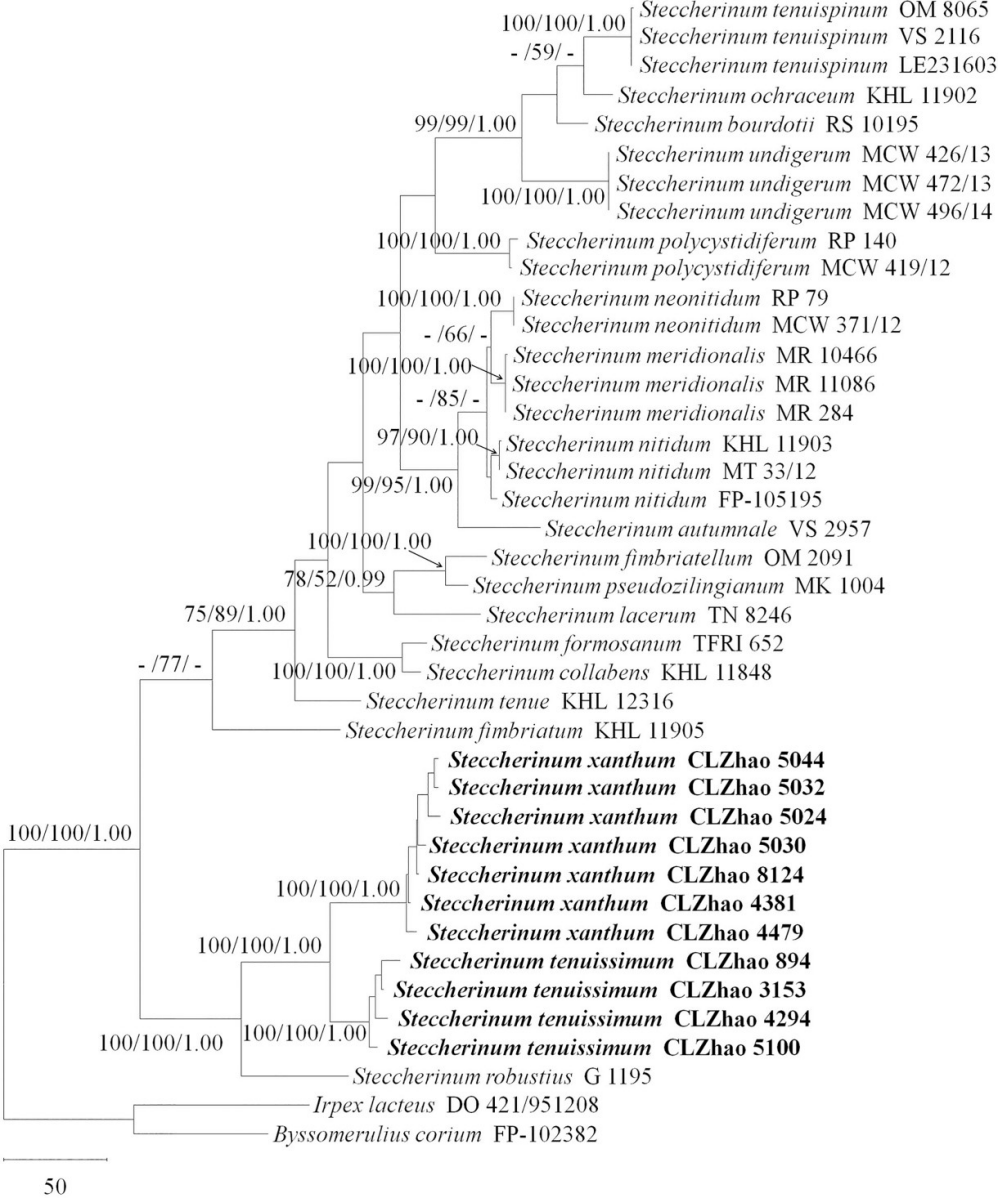
Maximum parsimony strict consensus tree illustrating the phylogeny of two new species and related species of *Steccherinum* based on ITS + nLSU sequences. Branches are labelled with maximum likelihood bootstrap values higher than 70%, parsimony bootstrap proportions higher than 50% and Bayesian posterior probabilities more than 0.95.

Sequencher 4.6 (GeneCodes, Ann Arbor, MI, USA) was used to edit the DNA sequences. The sequences were aligned in MAFFT 7 (http://mafft.cbrc.jp/alignment/server/) using the “G-INS-I” strategy and manually adjusted in BioEdit [12]. The sequence alignment was deposited in TreeBase (submission ID 27218). Sequences of *Heterobasidion annosum* (Fr.) Bref. and *Stereum hirsutum* (Willd.) Pers. obtained from GenBank was used as an outgroup to root trees following previous study [6] in the ITS + nLSU analysis (Fig 1), and *Byssomerulius corium* (Pers.) Parmasto and *Irpex lacteus* (Fr.) Fr. were used as an outgroup in the ITS + nLSU (Fig 2) analyses following previous study [8].

Maximum parsimony analyses were applied to the ITS + nLSU dataset sequences. The approaches used for the phylogenetic analysis followed previous study [13], and the tree construction procedure was performed in PAUP* version 4.0b10 [14]. All characters were equally weighted, and gaps were treated as missing data. Trees were inferred using the heuristic search option with TBR branch swapping and 1000 random sequence additions. Max-trees was set to 5000, branches of zero length were collapsed, and all parsimonious trees were saved. Clade robustness was assessed using a bootstrap (BT) analysis with 1000 replicates [29]. Descriptive tree statistics, including tree length (TL), consistency index (CI), retention index (RI), rescaled consistency index (RC), and homoplasy index (HI) were calculated for each maximum parsimony tree generated. The sequences were also analysed using maximum likelihood (ML) with RAxML-HPC2 through the Cipres Science Gateway (www.phylo.org) [30]. Branch support (BS) for the ML analysis was determined by 1000 bootstrap replicates.

MrModeltest 2.3 [31] was used to determine the best-fit evolution model for each dataset through Bayesian inference (BI). Bayesian inference was calculated with MrBayes 3.1.2, with a general time reversible (GTR+I+G) model of DNA substitution and gamma distribution rate variation across sites [32]. Four Markov chains were run for 2 runs from random starting trees for 800 thousand generations (Fig 1), for 1100 thousand generations (Fig 2) and trees were sampled every 100 generations. The first one-fourth of the generations were discarded as burn-in. A majority rule consensus tree of all the remaining trees was calculated. The branches were considered significantly supported if they received maximum likelihood bootstrap values (BS) >75%, maximum parsimony bootstrap values (BT) >75%, or Bayesian posterior probabilities (BPP) >0.95.

### Nomenclature acts

The electronic version of this article in Portable Document Format (PDF) in a work with an ISSN or ISBN will represent a published work according to the International Code of Nomenclature for algae, fungi, and plants, and hence the new names contained in the electronic publication of a PLOS article are effectively published under that Code from the electronic edition alone, so there is no longer any need to provide printed copies.

In addition, new names contained in this work have been submitted to MycoBank from where they will be made available to the Global Names Index. The unique MycoBank number can be resolved and the associated information viewed through any standard web browser by appending the MycoBank number contained in this publication to the prefix http://www.mycobank.org/MB/. The online version of this work is archived and available from the following digital repositories: PubMed Central and LOCKSS.

## Results

### Molecular phylogeny

The ITS + nLSU dataset (Fig 1) included sequences from 49 fungal specimens representing 45 species. The dataset had an aligned length of 1820 characters, of which 995 characters are constant, 202 are variable and parsimony-uninformative, and 623 are parsimony-informative. The maximum parsimony analysis yielded 2 equally parsimonious trees (TL = 4151, CI = 0.3404, HI = 0.6596, RI = 0.5438 and RC = 0.1851). The best model for the ITS + nLSU dataset estimated and applied in the Bayesian analysis was GTR+I+G (lset nst = 6, rates = invgamma, prset statefreqpr = dirichlet (1,1,1,1)). The Bayesian analysis and ML analysis resulted in a similar topology as the MP analysis, with an average standard deviation of split frequencies of 0.006728 (BI).

The phylogenetic tree (Fig 1) inferred from the ITS + nLSU sequences, demonstrated seven major clades for 45 sampled species in Polyporales. Two new *Steccherinum* species nested into the residual polyporoid clade. *Steccherinum xanthum* grouped with *S*. *tenuissimum* and was closely related to *S*. *robustius* (J. Erikss. & S. Lundell) J. Erikss.

The ITS + nLSU dataset (Fig 2) included sequences from 40 fungal specimens representing 21 species. The dataset had an aligned length of 2117 characters, of which 1587 characters are constant, 151 are variable and parsimony-uninformative, and 379 are parsimony-informative. The maximum parsimony analysis yielded 576 equally parsimonious trees (TL = 1322, CI = 0.551, HI = 0.449, RI = 0.798 and RC = 0.440). The best model for the ITS + nLSU dataset estimated and applied in the Bayesian analysis was GTR+I+G (lset nst = 6, rates = invgamma, prset statefreqpr = dirichlet (1,1,1,1)). The Bayesian analysis and ML analysis resulted in a similar topology as the MP analysis, with an average standard deviation of split frequencies of 0.009054 (BI).

The phylogenetic tree (Fig 2) inferred from the ITS + nLSU sequences had 19 species of *Steccherinum* and revealed that *Steccherinum tenuissimum* and *S*. *xanthum* were sister to *S*. *robustius* with high support (100% BP, 100% BS and 1.00 BPP). *Steccherinum tenuissimum* and *S*. *xanthum* formed a well-supported monophyletic lineage distinct from other *Steccherinum* species.

### Taxonomy

**Fig 3:**
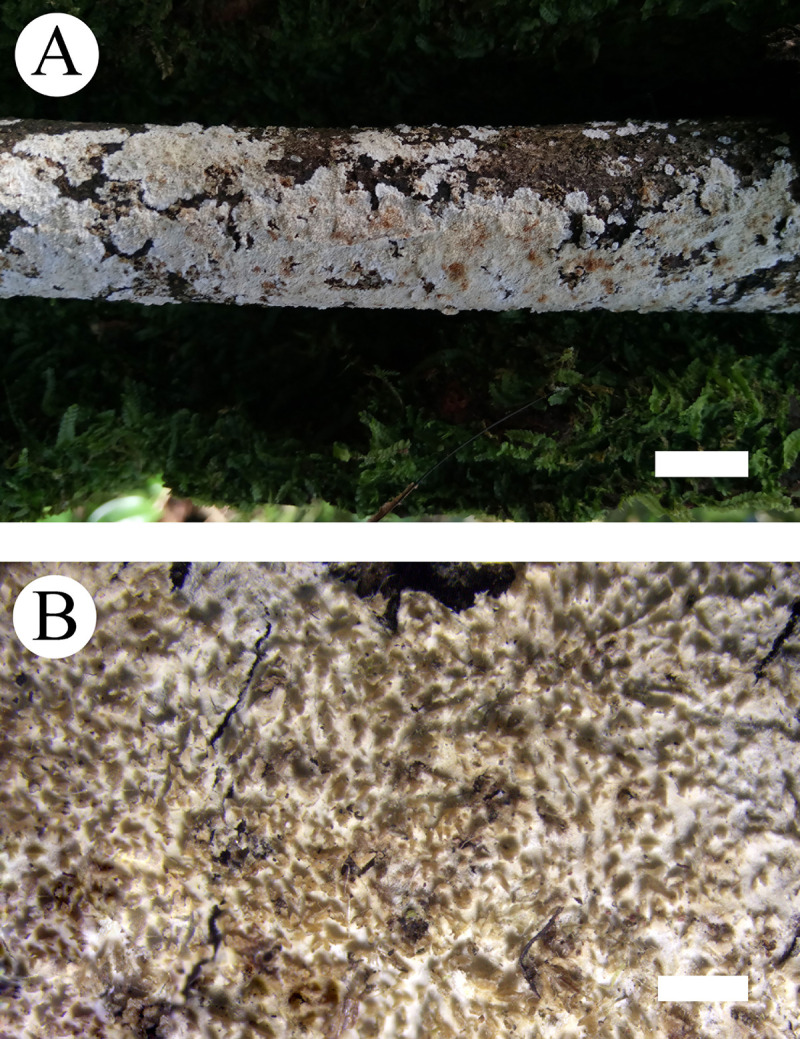
Basidiomata of *Steccherinum tenuissimum*. Bars: A = 1 cm, B = 1 mm (holotype).

**Fig 4:**
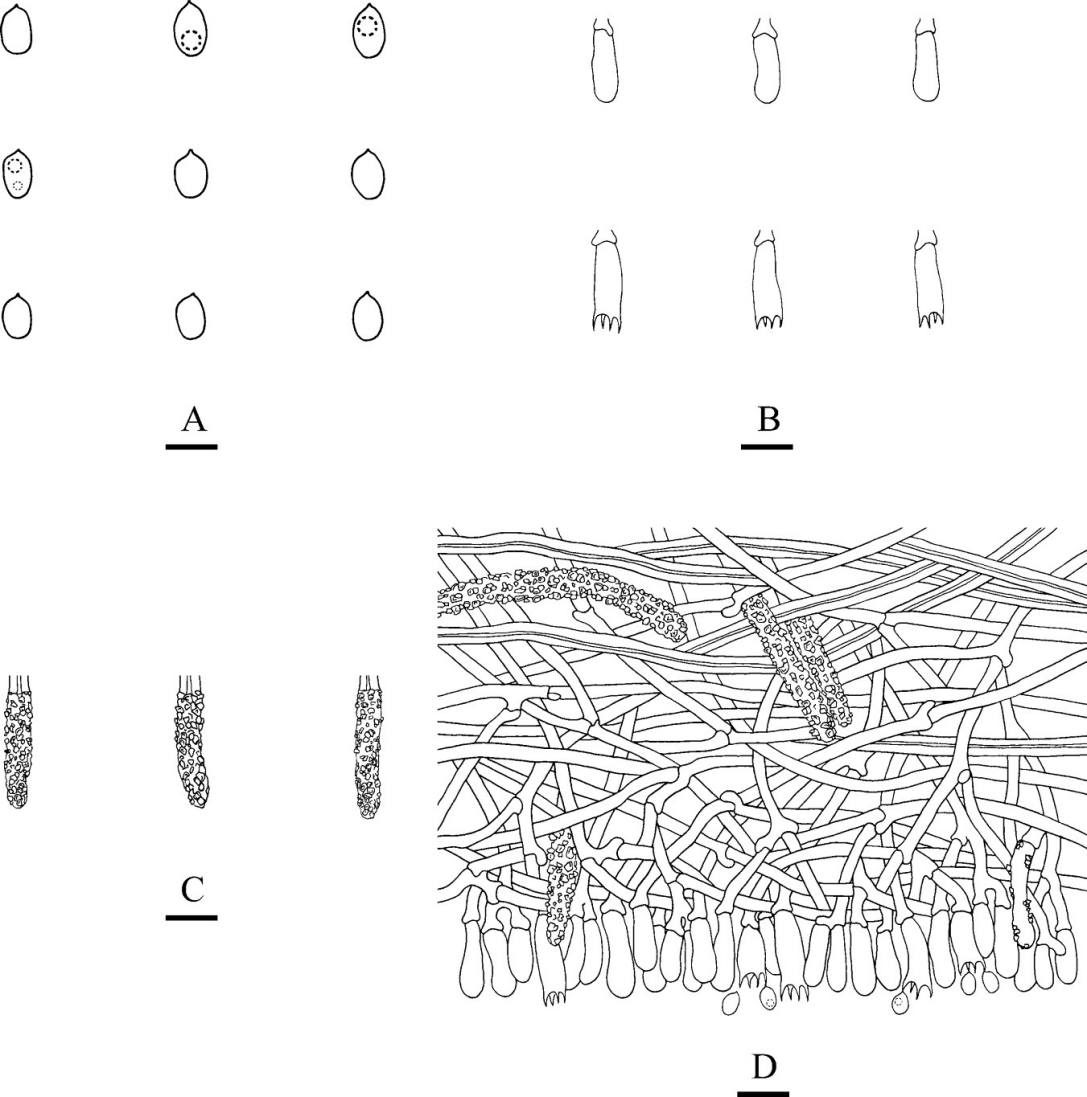
Microscopic structures of *Steccherinum tenuissimum* (drawn from the holotype). A: Basidiospores. B: Basidia and basidioles. C: Cystidia. D: A section of hymenium. Bars: A = 5 μm, B–D = 10 μm.

***Steccherinum tenuissimum*** C.L. Zhao & Y.X. Wu, sp. nov. Figs 3 and 4

MycoBank No.: MB 837795

Holotype: China. Yunnan Province, Pu’er, Laiyanghe National Forest Park, on fallen angiosperm branch, 30 September 2017, CLZhao 3153 (SWFC).

Etymology: *tenuissimum* (Lat.): referring to the relatively thin basidiomata.

Basidiomata: Annual, adnate, without odour or taste when fresh, becoming membranaceous up on drying, very thin, up to 20 cm long, 3 cm wide, 50–100 μm thick. Hymenial surface odontioid, with round aculei, 3–4 per mm, 0.2–0.5 mm long, white to cream when fresh, turning to cream to olivaceous buff upon drying.

Hyphal structure: Hyphal system dimitic, generative hyphae with clamp connections, colourless, thin-walled, branched, interwoven, 1.8–3.5 μm in diameter, IKI–, CB–; skeletal hyphae colourless, thick-walled, unbranched, 2.4–4.5 μm in diameter, IKI–, CB+; tissues unchanged in KOH.

Hymenium: Cystidia numerous, colourless, strongly encrusted, 22–39 × 4.5–6 μm; cystidioles absent. Basidia subclavate, with 4-sterigmata and basal clamp connections, 9.5–19 × 2.5–5.5 μm; basidioles dominant, in shape similar to basidia, but slightly smaller.

Spores: Basidiospores ellipsoid, colourless, thin-walled, smooth, with oil drops, IKI–, CB–, 3–5(–5.5) × 2–3.5 μm, L = 4.16 μm, W = 2.86 μm, Q = 1.40–1.52 (n = 120/4).

Additional specimens (paratypes) examined: China. Yunnan Province, Yuxi, Xinping County, Mopanshan National Forest Park, on fallen angiosperm branch, 16 January 2017, CLZhao 894 (SWFC); Xinping County, the Ancient Tea–Horse Road, on fallen angiosperm branch, 13 January 2018, CLZhao 5100 (SWFC); Pu’er, Jingdong County, Wuliangshan National Nature Reserve, on fallen branch of *Pinus*, 5 October 2017, CLZhao 4294 (SWFC).

**Fig 5:**
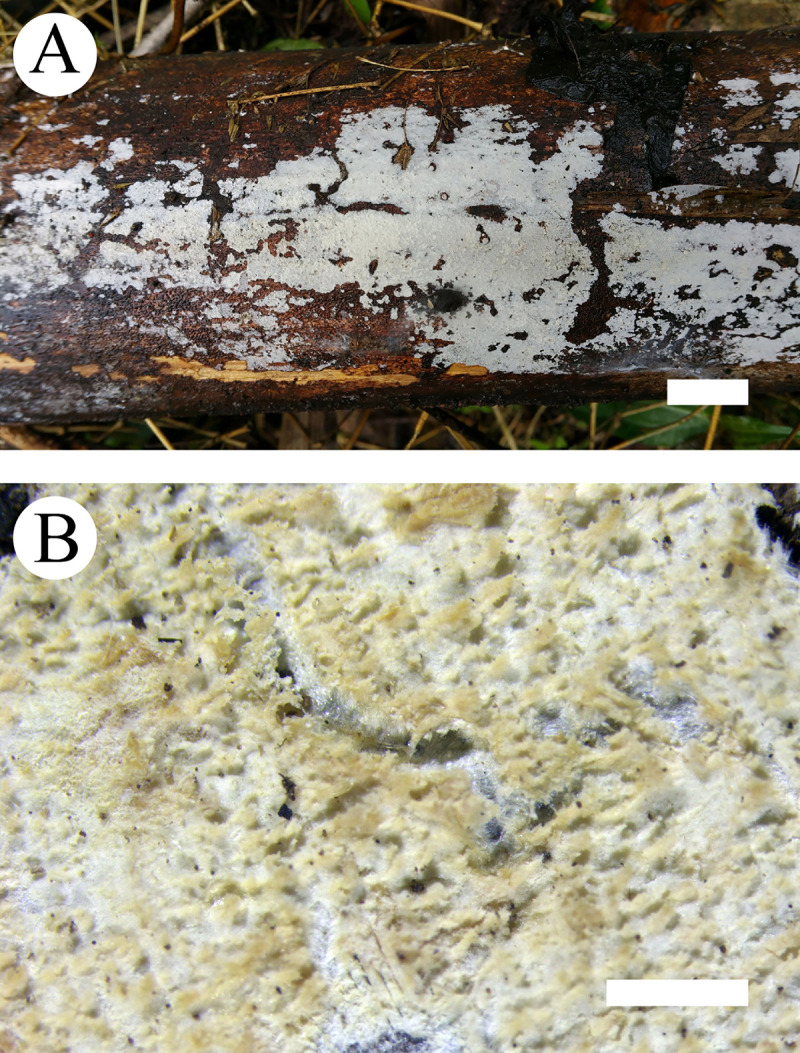
Basidiomata of *Steccherinum xanthum*. Bars: A = 1 cm, B = 1 mm (holotype).

**Fig 6:**
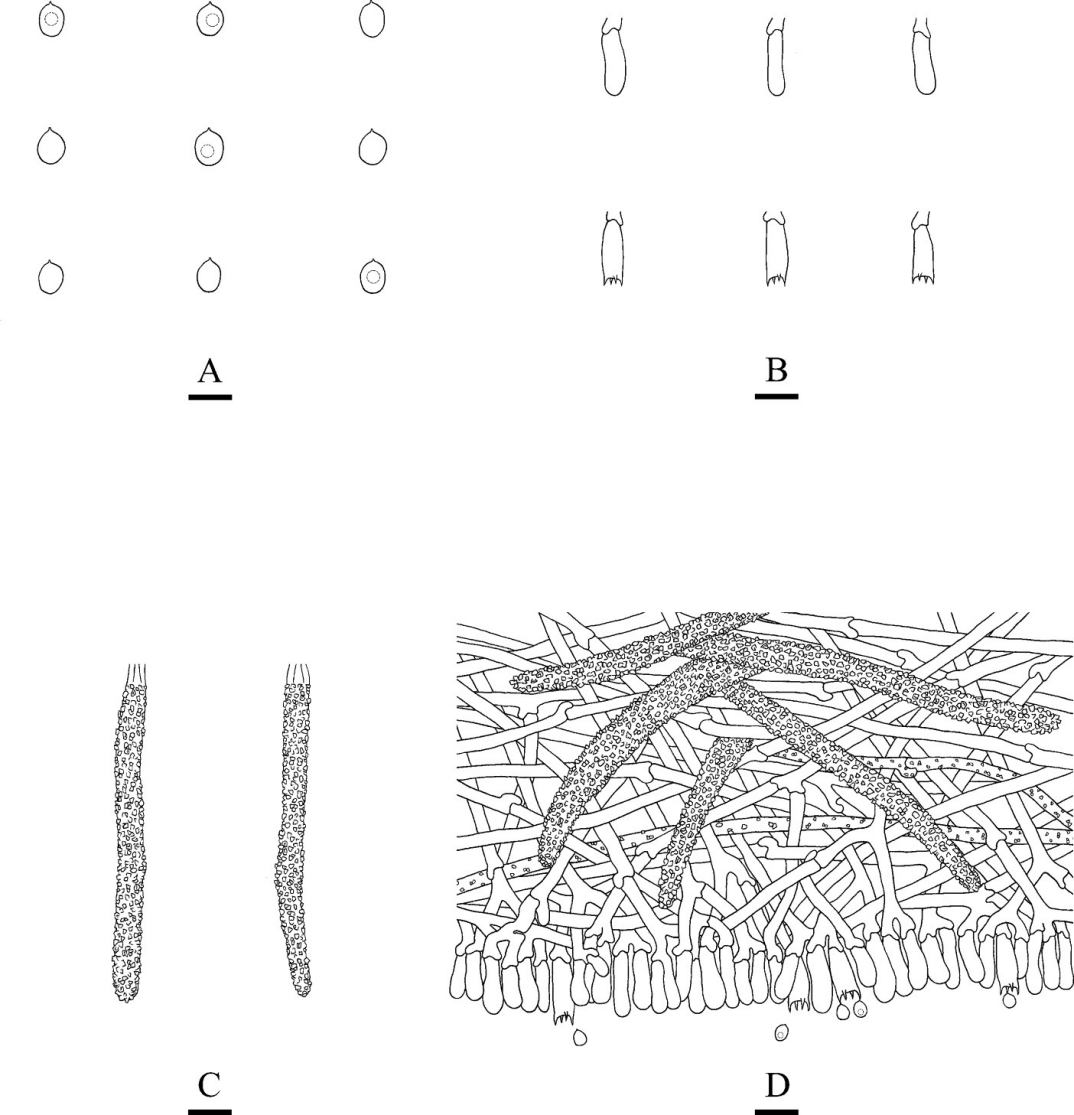
Microscopic structures of *Steccherinum xanthum* (drawn from the holotype). A: Basidiospores. B: Basidia and basidioles. C: Cystidia. D: A section of hymenium. Bars: A = 5 μm, B–D = 10 μm.

***Steccherinum xanthum*** C.L. Zhao & Y.X. Wu, sp. nov. Figs 5 and 6

MycoBank No.: MB 837796

Holotype: China. Yunnan Province, Pu’er, Jingdong County, Wuliangshan National Nature Reserve, on fallen angiosperm branch, 6 October 2017, CLZhao 4479 (SWFC).

Etymology: *xanthum* (Lat.): referring to the buff hymenial surface of the type specimen.

Basidiomata: Annual, resupinate, adnate, without odour or taste when fresh, becoming membranaceous up on drying, up to 10 cm long, 4 cm wide, 100–200 μm thick. Hymenial surface odontioid, with round aculei, 5–6 per mm, 0.1–0.3 mm long, white to cream when fresh, turning buff to yellow upon drying.

Hyphal structure: Hyphal system monomitic, generative hyphae with clamp connections, colourless, thin-walled, branched, covered by crystals, interwoven, 2–4.5 μm in diameter; IKI–, CB–; tissues unchanged in KOH.

Hymenium: Cystidia numerous, strongly encrusted in the apical part, 35.5–125 × 5–9 μm; cystidioles absent. Basidia clavate, with 4-sterigmata and basal clamp connections, 10–19.3 × 3–5.2 μm, basidioles dominant, in a shape similar to basidia, but slightly smaller.

Spores: Basidiospores ellipsoid, colourless, smooth, thin-walled, with oil drops, IKI–, CB–, 2.7–5(–5.6) × 2–3.9(–4.4) μm, L = 3.48 μm, W = 2.63 μm, Q = 1.25–1.41 (n = 240/9).

Additional specimens (paratypes) examined: China. Yunnan Province, Pu’er, Zhenyuan County, Heping town, Jinshan Forest Park, on fallen angiosperm trunk, 12 January 2018, CLZhao 5024, 5032, 5044 (SWFC); 21 August 2018, CLZhao 8124 (SWFC); on fallen angiosperm branch 12 January 2018, CLZhao 5030 (SWFC); Jingdong County, Wuliangshan National Nature Reserve, on fallen angiosperm trunk, 6 October 2017, CLZhao 4381 (SWFC).

## Discussion

In the present study, two new species, *Steccherinum tenuissimum* and *S*. *xanthum* spp. nov., are described based on phylogenetic analyses and morphological characters.

Phylogenetically, seven clades were found in Polyporales: the residual polyporoid clade, the phlebioid clade, the antrodia clade, the tyromyces clade, the fragiliporia clade, the core polyporoid clade and the gelatoporia clade [6, 27]. According to our result based on the combined ITS + nLSU sequence data (Fig 1), two new species are nested into the residual polyporoid clade with strong support (100% BS, 100% BP, 1.00 BPP).

*Steccherinum tenuissimum* and *S*. *xanthum* were closely related to *S*. *robustius* based on rDNA sequences (Fig 2). However, morphologically *S*. *robustius* differs from the two new species by having a reddish orange to pale orange or brown hymenial surface and pale yellowish cystidia [2]. *S*. *tenuissimum* differs from *S*. *xanthum* by the cream to olivaceous hymenial surface and a dimitic hyphal system.

Geographically *Steccherinum subglobosum* H.S. Yuan & Y.C. Dai and *S*. *subulatum* H.S. Yuan & Y.C. Dai were described as new to science in P.R. China, but morphologically, *S*. *subglobosum* differs in its effuse-reflexed to pileate basidiomata with velutinate to tomentose hymenial surface and subglobose basidiospores (3.9–4.6 × 3.3–3.9 μm), *S*. *subulatum* differs from the two new taxa in the resupinate to effuse-reflexed basidiomata with longer hymenophore spines [33].

Wood-rotting fungi are an extensively studied group of Basidiomycota [2, 5, 6, 10, 34–37], but Chinese wood-rotting fungal diversity is still not well known, especially in the subtropics and tropics. Many recently described taxa of wood-rotting fungi are from subtropical and tropical areas in China [38–42]. The two new species in the present study are also from the subtropics. It is possible that new taxa will be found after further investigations and molecular analyses.
